# Enrichment of bacteria and alginate lyase genes potentially involved in brown alga degradation in the gut of marine gastropods

**DOI:** 10.1038/s41598-018-38356-y

**Published:** 2019-02-14

**Authors:** Michihiro Ito, Kotaro Watanabe, Toru Maruyama, Tetsushi Mori, Kentaro Niwa, Seinen Chow, Haruko Takeyama

**Affiliations:** 1 Research Organization for Nano & Life Innovation, Waseda University, 513 Wasedatsurumaki-cho, Shinjuku, Tokyo 162-0041 Japan; 2 Tropical Biosphere Research Center, University of the Ryukyus, 1 Senbaru, Nishihara, Okinawa 903-0213 Japan; 3 Department of Life Science and Medical Bioscience, Waseda University, 2-2 Wakamatsu-cho, Shinjuku, Tokyo 162-8480 Japan; 4 International Center for Science and Engineering Programs, Waseda University, 3-4-1 Okubo, Shinjuku-ku, Tokyo 169-8555 Japan; 5 Department of Biotechnology and Life Science, Tokyo University of Agriculture and Technology, 2-24-16 Naka-cho, Koganei, Tokyo 184-8588 Japan; 6 National Research Institute of Fisheries Science, 2-12-4 Fukuura, Kanazawa, Yokohama, Kanagawa 236-8648 Japan; 7 Institute for Advanced Research of Biosystem Dynamics, Waseda University, 2-2 Wakamatsu-cho, Shinjuku, Tokyo 162-8480 Japan; 8 Computational Bio Big-Data Open Innovation Laboratory, AIST-Waseda University, 3-4-1 Okubo, Shinjuku, Tokyo 169-0072 Japan

## Abstract

Gut bacteria of phytophagous and omnivorous marine invertebrates often possess alginate lyases (ALGs), which are key enzymes for utilizing macroalgae as carbon neutral biomass. We hypothesized that the exclusive feeding of a target alga to marine invertebrates would shift the gut bacterial diversity suitable for degrading the algal components. To test this hypothesis, we reared sea hare (*Dolabella auricularia*) and sea snail (*Batillus cornutus*) for two to four weeks with exclusive feeding of a brown alga (*Ecklonia cava*). Pyrosequencing analysis of the gut bacterial 16S rRNA genes revealed shifts in the gut microbiota after rearing, mainly due to a decrease in the variety of bacterial members. Significant increases in six and four 16S rRNA gene phylotypes were observed in the reared sea hares and sea snails, respectively, and some of them were phylogenetically close to known alginate-degrading bacteria. Clone library analysis of PL7 family ALG genes using newly designed degenerate primer sets detected a total of 50 ALG gene phylotypes based on 90% amino acid identity. The number of ALG gene phylotypes increased in the reared sea hare but decreased in reared sea snail samples, and no phylotype was shared between them. Out of the 50 phylotypes, 15 were detected only after the feeding procedure. Thus, controlled feeding strategy may be valid and useful for the efficient screening of genes suitable for target alga fermentation.

## Introduction

There have been recent developments in the use of marine macroalgae for biofuel production because of their high productivity, high abundance in nations bordering the sea, and compatibility with food resources^1–3^. The presently available procedures for fuel ethanol production from macroalgae components employ biological processes consolidated in microbial platforms^3–6^. The concept is promising; for example, Wargacki *et al*.^5^ achieved a yield of 0.281 weight ethanol/weight dry macroalgae using genetically modified *Escherichia coli* cells. However, their method has not yet been put into practical use. Improving each enzymatic process would greatly facilitate the development of fermentation technology using marine macroalgae.

Brown algae have the highest productivity among marine macroalgae^7^. They contain polysaccharides such as alginate, fucoidan, cellulose, laminaran, and mannitol, among which alginate is the predominant structural polysaccharide constituting 10‒40% of dry weight^8^. No microorganism has been reported to directly ferment alginate into ethanol. Therefore, alginate degradation into monosaccharides is a key step for the fermentation of brown algae. Alginate is a linear homopolymer in which two monosaccharides, β-D-mannuronic acid and α-L-guluronic acid, are concatenated randomly^6^. The structure thus contains blocks of poly-mannuronic acid [poly (M)], blocks of poly-guluronic acid [poly (G)], and hetero-polymeric blocks consisting of both monosaccharides [poly (M-G)]^9^. The relative abundance of each block in brown algae such as *Ecklonia cava* and *Laminaria japonica* varies depending on algal parts and season^10–13^.

Alginates are degraded into smaller polymers or even monosaccharides by a group of alginate lyase (ALG) that catalyze the β-elimination reaction of the glucosidic bonds between the monosaccharide residues^9^. To date, a number of alginate-degrading bacteria have been isolated, and their ALG genes have been identified^9,14–17^. Several marine invertebrates also possess ALG genes^9^. These ALG genes are classified into seven (PL5, PL6, PL7, PL14, PL15, PL17, and PL18) polysaccharide-degrading enzyme families (PLs) based on their primary structures^18^. PL7 is the major family among the ALGs identified to date. PL7 ALGs generally degrade alginates into oligosaccharides in an endolytic manner^19,20^, whereas an ALG, AlyA5 from *Zobellia galactanivorans*, degrades alginates in an exolytic manner^21^. Each ALG enzyme has a different substrate specificity. Endolytic ALGs can be classified into three types based on the preference of the attacking sites; some ALGs prefer a [poly (M)] structure, whereas others prefer [poly (G)] or [poly (M-G)]^19^. Exolytic ALGs also have a preference regarding the length of the polymers^22^.

The microbiota in the gut of animals are linked with host feed digestion. Cellulose-degrading microbiota inhabit the gut of lower termites that eat wood biomass^23,24^. Tannin-protein-complex-degrading enterobacteria were detected in the feces of koala that eat eucalypts^25^. Similarly, several bacterial alginate degraders were found in the gut microbiota of brown algae-eating marine invertebrates such as sea snails^26^, sea urchins^27^, and abalones^28–32^. Therefore, the gut microbiota of phytophagous or omnivorous marine invertebrates may be suitable gene resources for the degradation of macroalgae components. Tanaka *et al*.^29^ reported that the gut microbiota of captive abalones changed coincidently with a change in diet, suggesting the feasibility of controlling gut microbiota of marine invertebrates by feeding. Modifications of gut microbiota by diet change have also been reported in other animals such as termites, land snails and humans^33–36^. We hypothesized that under captive condition by feeding a single algal species, the gut microbiota of marine invertebrates would shift to a state more adaptive to digesting the algal species. The procedure for rearing marine invertebrates with a brown alga could result in the gut microbiota that habour genes necessary for degradation of the algal alginate polymers.

In this study, the concept of an “*in vivo* enrichment strategy” was tested through a rearing experiment using two phytophagous gastropods, the sea hare *Dolabella auricularia* and the sea snail *Batillus cornutus*, as host animals and a brown alga, *Ecklonia cava*, as their sole food source. These two marine gastropods are known to feed on various macroalgae^37–41^. The midgut glands in the two gastropods and the gastric teeth in the sea hare have alginate-degrading activities^42–44^, and ALGs were isolated from the homogenates of their midgut glands^42,44,45^. Three bacterial isolates from *B. cornutus* guts were reported to degrade algal components such as alginate and laminaran^26^; however, no study on alginate degrading bacteria has been performed for *D. auricularia*. No literature has reported on the gut microbial communities of the two gastropods so far. Since the two species are phytophagous, we expected bacterial alginate degraders to be resident in their guts. The effects of the rearing experiment were evaluated by comparing 16S rDNA and PL7 ALG gene profiles between wild and reared individuals.

## Results

### Reared Animals

All individuals used for rearing experiment (seven for the sea hare (SH) *D. auricularia* and six for the sea snail (SS) *B. cornutus*) survived until the end of experiment. Daily active feeding and defecation were observed in all individuals. Weight loss was observed in five of seven SH individuals and one of six SS individuals. SH is hermaphroditic and was observed to frequently lay egg mass in captivity, which might be responsible for the weight loss. Thus, the captive environment may not be inappropriate and stressful for the animals.

### Shift of the Gut Microbiota During the Rearing Experiment

A total of 4,035-24,907 sequence reads of the 16S rDNA V1-V2 region that passed all our quality control procedure were obtained (Table S1).

**Figure 1:**
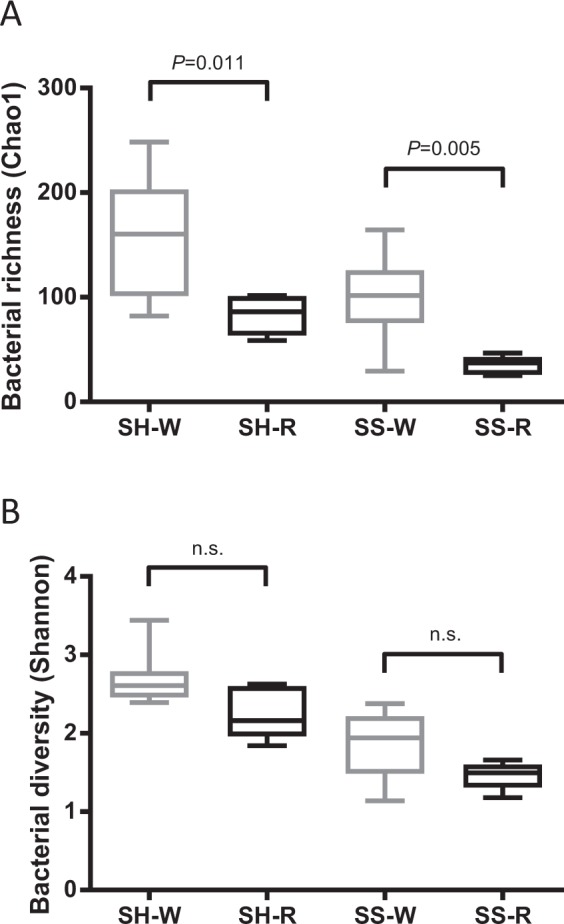
Box plot representation of (**A**) richness indices (Chao1) and (**B**) diversity indices (Shannon) of 16S rRNA gene phylotypes in the gut microbiota of sea hares (SH) and sea snails (SS). The numbers of phylotypes in each sample were obtained from 4,000 sequence reads. Medians (solid lines) are shown in each box. The bottom and top of the box indicate the 25th and 75th percentiles, respectively. The bottom and top of the error bars represent the minimum and maximum values, respectively. W, wild samples; R, reared samples; n.s., not significant.

The indices of 16S rRNA gene phylotype richness (Chao1) and diversity (Shannon) were compared between wild and reared individuals. Because the richness indices depend on the number of sequence reads, we used the same number of reads (4,000 reads) extracted from each data set by random sampling. Significantly lower bacterial richness was observed in the reared SH and SS samples than in the wild samples (*p* < 0.05) (Fig. 1A). Not significant but lower bacterial diversity was observed in the reared SH and SS samples (SH, 1.84-2.63; SS, 1.18-1.66) than in the wild samples (SH, 2.39-3.44; SS, 1.14-2.38) (Fig. 1B; Table S1).

**Figure 2:**
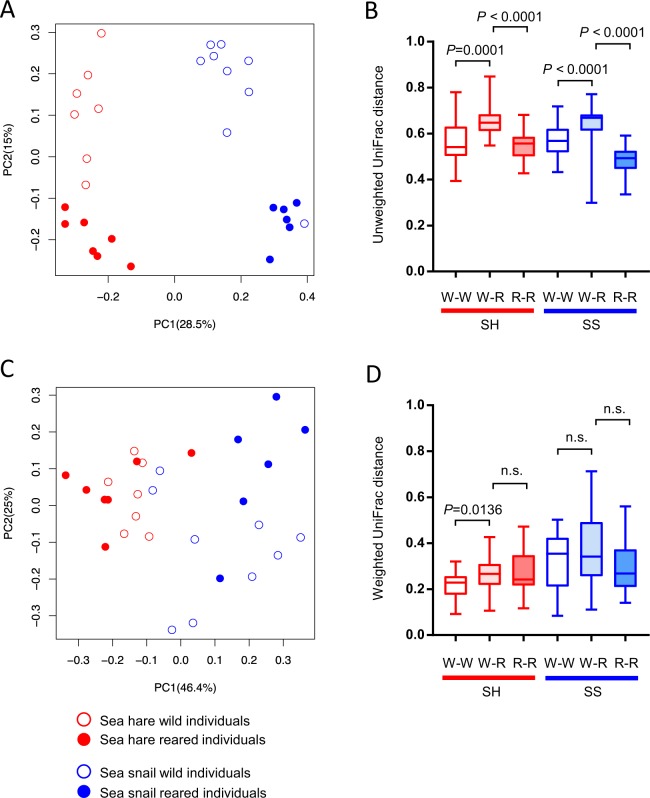
UniFrac distance analysis of 16S rRNA gene compositions in the gut microbiota of sea hares (SH) and sea snails (SS). (**A** and **C**) Principal coordinate analysis (PCoA) of 16S rRNA gene compositions based on unweighted (**A**) and weighted (**C**) UniFrac distances. Because of the low explained variance of the first two components in the panel A (43.5% in total), we confirmed the preservation of inter-sample distances in the two components of the PCoA (Fig. S2), following the literature by Hervé *et al*.^65^ (**B** and **D**) UniFrac distance values between wild (W) and reared (R) samples (W-R) and within the same sample type (W-W, R-R). Medians (solid lines) are shown in each box. The bottom and top of the box indicate the 25th and 75th percentiles, respectively. The bottom and top of the error bars represent the minimum and maximum values, respectively. n.s., not significant.

The similarity of bacterial community structures in the gut microbiota was analyzed with the extracted 4,000 reads using principal coordinate analysis (PCoA) based on unweighted and weighted UniFrac distances (UDs) (Fig. 2A,C)^46,47^. The unweighted UD reflects differences only in community members (qualitative), whereas weighted UD reflects differences in both community members and their relative abundance within the datasets (quantitative). The unweighted PCoA in the present study illustrated (i) that the plots of SH and SS gut microbiota were distributed separately from each other and (ii) that the plots of wild and reared individuals were also distributed separately with respect to both gastropod species. We compared the unweighted and weighted UDs within wild individuals, within reared individuals and between wild and reared individuals for each gastropod (Fig. 2C,D). Larger UD implies higher variability of microbial community structure within and between sample types. If statistically significant difference is detected between any pair of the three differences, it can be considered that our rearing experiment significantly affected the overall structure of gut microbiota composition^48^. The unweighted UDs between wild and reared individuals were significantly different from those within the same sample types (Fig. 2B). These data suggested that all four sample types were qualitatively different from one another. The PCoA and distance analysis based on weighted UDs did not detect such notable difference between the wild and reared SH samples (Fig. 2B,D).

**Figure 3:**
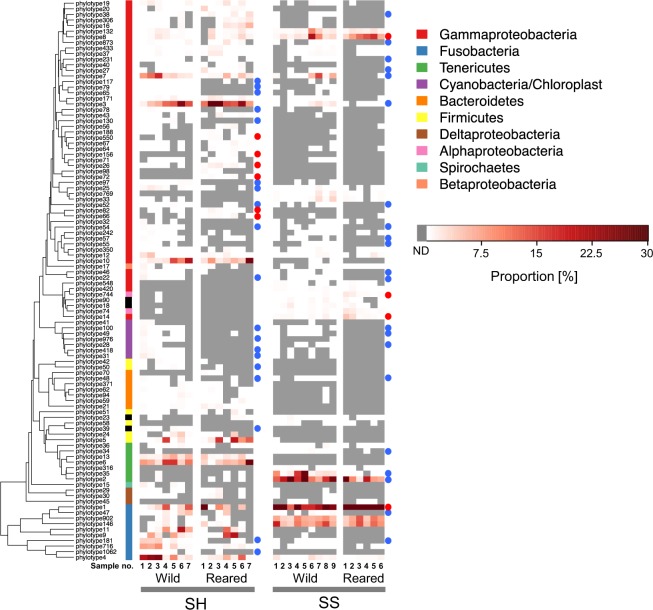
Distribution and abundance of 16S rRNA gene phylotypes in the gut microbiota of sea hares (SH) and sea snails (SS). Red and blue circles in SH and SS indicate the phylotypes with significantly higher and lower abundances in the reared samples, respectively. ND, not detected.

We constructed a phylogenetic tree with the abundance values of the top 100 16S rRNA gene phylotypes (Fig. 3). In general, the numbers of phylotypes were lower in the reared samples. However, we found phylotypes with significantly higher abundance in the reared SH (phylotypes 26, 66, 72, 82, 156, and 550) and SS (phylotypes 1, 8, 74, and 744) samples than in the wild samples. Phylotypes 26, 156, and 550 showed the highest identities (95‒96%) to 16S rRNA genes of the bacteria belonging to the genus *Psychromonas* (Table 1). Several reported bacterial isolates of the genus *Psychromonas* possess alginate-degrading activities^49,50^; we found that several genomic contigs or scaffolds of three *Psychromonas* strains (*Psychromonas* sp. SP041, *P. arctica* DSM14288, and *P. hadalis* ATCC BAA-638) contain alginate lyase (ALG) gene candidates in the NCBI Reference sequence database (e.g., RefSeq accession no. NZ_CBRF010000088.1 and NZ_ATUO01000052.1). Phylotypes 8, 66, 72, and 82 exhibited the highest identities (92–100%) to the strains of the genus *Vibrio* (Table 1), a taxon that often possesses one or more alginate lyase genes in their genome sequence^20,51,52^. Phylotype 8 showed 100% identity to the 16S rRNA gene of *Vibrio neonatus*, a strain with an alginate-degrading activity isolated from the intestine of farmed abalone (Table 1)^30^. Phylotype 1 showed 97% identity to a strain of the genus *Psychrilyobacter* (Table 1). The 16S sequences close to this genus have been detected in marine molluscs such as octopus (GenBank accession no. HM007338) and oyster^53^. No strain of this genus has been reported to possess alginate-degrading activities. Top hits for phylotypes 74 and 744 were the known proteobacterial 16S rRNA gene, but the identities were very low (86 and 84%, respectively) (Table 1).

**Table 1:** 16S rRNA gene phylotypes with significant change in relative abundance of gut microbiota of sea hares and sea snails. ^a^The numbers correspond to the phylotype numbers in Fig. 3.

Phylotype no.^a^	Description	Closest relatives in the database	GenBank Accession no.	% Nucleotide identity
26	Higher relative abundance in sea hare reared samples compared to its wild samples	*Psychromonas* sp. FS11–3	JQ799970	96 (270/281)
66		*Vibrio* sp. Sydb5	KC462976	98 (254/257)
72		*Vibrio splendidus*	HE584792	92 (258/280)
82		*Vibrio* sp. TJD753	DQ993344	97 (276/282)
156		*Psychromonas profunda*	FJ752873	95 (268/280)
550		*Psychromonas* sp. KJF6–3	JQ800095	96 (271/280)
1	Higher relative abundance in sea snail reared samples compared to its wild samples	*Psychrilyobacter* sp. STAB704	JF825448	97 (272/280)
8		*Vibrio neonatus*	HE584778	100 (280/280)
74		Alpha proteobacterium PR-1	KJ442651	86 (244/282)
744		*Litorimonas cladophorae*	JX174422	84 (242/285)
25	Lower relative abundance in sea hare reared samples compared to its wild samples	*Vibrio* sp. r32	AB470935	96 (269/280)
31		bacterium WHC1–2	JQ269270	92 (259/280)
39		Mollicutes bacterium HR1	CP009415	84 (237/280)
50		*Clostridium formicaceticum*	HF679208	82 (233/282)
65		*Photobacterium* sp. HM6–53	AB525425	100 (280/280)
78		*Vibrio tapetis*	Y08430	97 (273/280)
79		*Photobacterium* sp. S-7	DQ978988	99 (279/280)
97		bacterium w3cb2	DQ416659	98 (275/279)
117		*Photobacterium* sp. B2–26	JX134459	96 (271/280)
130		Vibrionaceae bacterium C16	DQ005883	98 (239/242)
418		bacterium EA10-B11–3	JF418022	95 (237/248)
976		bacterium WHC1–2	JQ269270	94 (265/280)
1062		*Propionigenium maris*	Y16800	96 (267/278)
22	Lower relative abundance in sea hare and sea snail reared samples compared to their wild samples	marine gamma proteobacterium HTCC 2246	AY386337	99 (271/273)
48		*Lutimonas* sp. PAORIC-13	KP030835	97 (273/280)
52		*Vibrio* sp. Mj207	GQ454968	98 (277/280)
54		*Paramoritella sediminis*	JQ672626	100 (267/267)
100		bacterium EA10-B11–3	JF418022	96 (240/248)
181		*Propionigenium maris*	Y16800	96 (269/280)
3	Lower relative abundance in sea snail reared samples compared to its wild samples	*Vibrio atlanticus*	KF994032	100 (280/280)
7		*Vibrio* sp. VibP-Oc-138	KF577039	100 (280/280)
27		*Vibrio* sp. V759	DQ146990	97 (272/280)
28		bacterium WHC1–2	JQ269270	95 (268/281)
34		*Mycoplasma vulturii*	AY191226	83 (237/284)
35		*Mycoplasma vulturii*	AY191226	85 (241/283)
38		*Vibrio hippocampi*	FN421434	99 (278/280)
46		bacterium IMCC8485	KJ492091	96 (271/280)
47		*Psychrilyobacter* sp. STAB703	JF825447	97 (273/280)
49		*Vibrio* sp. VibP-Oc-120	KF577036	89 (251/280)
55		*Shewanella* sp. EK8B	LC053422	100 (280/280)
57		*Shewanella* sp. NBRC 101066	AB681366	99 (279/280)
171		*Vibrio* sp. L-2–6	AB550535	99 (261/263)
231		*Vibrio* sp. V759	DQ146990	98 (275/280)
316		*Mycoplasma vulturii*	AY191226	84 (239/283)
873		*Vibrio* sp. MWB30	EU130466	99 (278/280)

There were a number of phylotypes whose abundance was significantly lower in the reared SH and SS samples than in the wild samples, respectively (Table 1, Fig. 3). Such phylotypes were taxonomically distributed over the phyla Fusobacteria, Bacteroidetes, Cyanobacteria, Tenericutes, and Proteobacteria. Several phylotypes belonging to *γ*-proteobacteria exhibited relatively high identities to known alginate-degrading bacterial genera such as *Vibrio, Photobacterium*, and *Shewanella*^9,17^.

### Shift of PL7 Family Alginate Lyase Gene Profiles During Rearing

To investigate PL7-family ALG gene profiles in the gut microbiota, we used newly designed degenerate primers targeting two conserved regions, strands A3 and A4 (Fig. S1). Because the range of the expected amplicon size (300‒650 bp) exceeded the range suitable for 454 sequencing (less than 150 bp is recommended by the manufacturer), the ALG gene profiles in gut microbiota were investigated with clone library analysis. Of 1,772 amplicons cloned, 933 sequences showed similarities to known ALG genes. Sequences with similarities to known ALG genes were found in all metagenomic samples except one wild SS individual. The 933 sequences were classified into 50 ALG phylotypes based on 90% amino acid identity.

**Figure 4:**
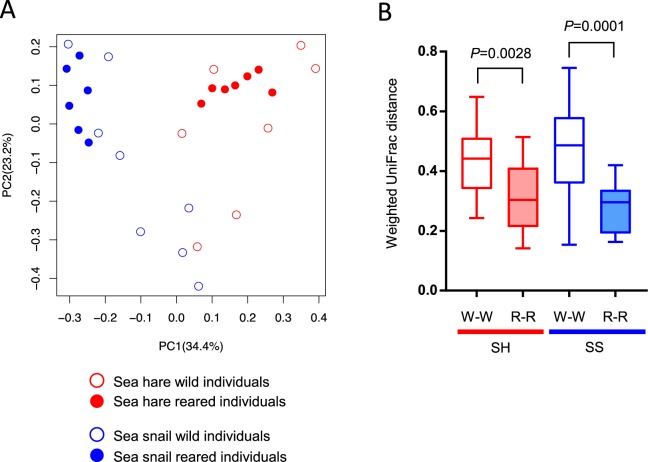
UniFrac distance analysis of PL7 alginate lyase gene compositions in the gut microbiota of sea hares (SH) and sea snails (SS). (**A**) Principal coordinate analysis (PCoA) of PL7 alginate lyase gene compositions based on weighted UniFrac distances. (**B**) Weighted UniFrac distance values within the same sample type. Medians (solid lines) are shown in each box. The bottom and top of the box indicate the 25th and 75th percentiles, respectively. The bottom and top of the error bars represent the minimum and maximum values, respectively. n.s., not significant.

**Figure 5:**
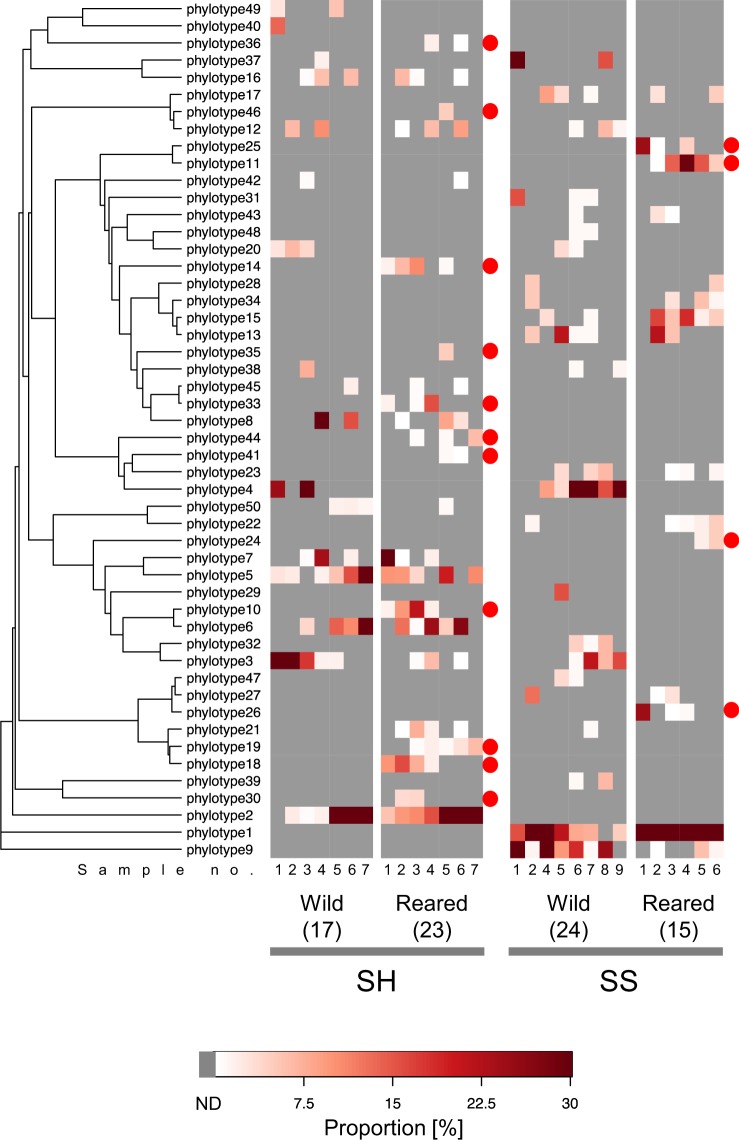
Distribution and abundance of PL7 alginate lyase gene phylotypes in the gut microbiota of sea hares (SH) and sea snails (SS). The numbers of phylotypes detected in each sample type are shown in parenthesis. Red circles indicate the phylotypes detected only in reared host animals.

The similarity of PL7 ALG gene profiles was evaluated using weighted PCoA (Fig. 4A). The plots of the reared SH and SS samples were each closely clustered, whereas those of the wild samples were dispersed. The weighted UDs between reared individuals were significantly lower than those between wild individuals for both two gastropods (Fig. 4B). The distribution and abundance of the 50 ALG phylotypes are illustrated in Fig. 5. For SH, we found 17 and 23 phylotypes in the wild and reared samples, respectively, among which 11 phylotypes were common. None of the phylotypes detected in the reared SH samples were found in the reared SS samples. With respect to the SS samples, 24 and 15 phylotypes were found from the wild and reared samples, respectively. The 15 phylotypes for the reared SS samples were not found in any SH sample. Out of the 50 phylotypes, 11 and 4 were SH reared- and SS reared-specific, respectively.

The 50 representative sequences of ALG gene phylotypes were subjected to a BLAST search against the nr database (Table 2). All of the sequences showed the highest identities to the sequences of the phylum Proteobacteria, and 42 out of 50 BLAST-top-hit sequences were of the genus *Vibrio*. The other sequences were phylogenetically closer to those of the genera *Agarivorans* (phylotypes 29 and 37), *Psychromonas* (phylotypes 30, 36, 39, and 46), and *Microbulbifer* (phylotypes 40 and 49). The 50 phylotypes shared 58‒99% identities with known ALG genes in the database in 112‒195 amino acids; 20 phylotypes showed ≥ 90% identities, and the other 30 showed only 58‒89%.

**Table 2:** Homologous proteins of representative sequences of 50 alginate lyase gene phylotypes from gut microbiota of sea hares and sea snails. ^a^The numbers correspond to the phylotype numbers in Fig. 5. ^b^SH, sea hare; SS, sea snail; W, wild individuals; R, reared individuals.

Phylotype no^a^	Origin of sequences^b^	Homologous proteins			
		Length	Host organism	GenBank or RefSeq Accession no.	% Amino-acid identity
1	SS-W, SS-R	323	*Vibrio halioticoli*	WP_023403960	83 (143/172)
2	SH-W, SH-R	318	*Vibrio breoganii*	WP_017032122	65 (113/174)
3	SH-W, SH-R, SS-W	380	*Vibrio* sp. JCM19236	WP_017242584	96 (170/178)
4	SH-W, SS-W	286	*Vibrio* sp. A9m	BAH79131	96 (107/112)
5	SH-W, SH-R	303	*Vibrio* sp. JCM 19236	GAM67127	60 (105/175)
6	SH-W, SH-R	345	*Vibrio* sp. J2–12	WP_050644410	96 (176/183)
7	SH-W, SH-R	347	*Vibrio hyugaensis*	WP_045499699	59 (109/185)
8	SH-W, SH-R	584	*Vibrio* sp. J2–29	WP_048616498	93 (150/162)
9	SS-W, SS-R	323	*Vibrio halioticoli*	WP_023403960	86 (149/174)
10	SH-R	345	*Vibrio cyclitrophicus*	WP_016799450	99 (178/179)
11	SS-R	302	*Vibrio halioticoli*	WP_039970820	56 (69/123)
12	SH-W, SH-R, SS-W	669	*Vibrio halioticoli*	WP_023404110	99 (191/192)
13	SS-W, SS-R	628	*Vibrio ezurae*	WP_021713707	68 (100/147)
14	SH-R	318	*Vibrio cyclitrophicus*	WP_010430034	99 (156/157)
15	SS-W, SS-R	628	*Vibrio ezurae*	WP_021713707	66 (103/157)
16	SH-W, SH-R	464	*Vibrio* sp.	WP_052879903	96 (165/171)
17	SS-W, SS-R	669	*Vibrio ezurae*	WP_021714326	92 (179/195)
18	SH-R	522	*Vibrio cyclitrophicus*	WP_016785028	99 (164/165)
19	SH-R	522	*Vibrio splendidus*	WP_061032705	95 (153/161)
20	SH-W, SS-W	323	*Vibrio breoganii*	WP_026026118	92 (109/119)
21	SH-R, SS-W	522	*Vibrio splendidus*	WP_061032705	99 (160/161)
22	SS-W, SS-R	346	*Vibrio diazotrophicus*	WP_042489980	78 (102/131)
23	SS-W, SS-R	283	*Vibrio* sp. J2–26	WP_050712693	58 (85/146)
24	SS-R	347	*Vibrio hyugaensis*	WP_045499699	69 (127/183)
25	SS-R	302	*Vibrio halioticoli*	WP_039970820	59 (82/140)
26	SS-R	521	*Vibrio halioticoli*	WP_023403957	90 (145/161)
27	SS-W, SS-R	521	*Vibrio halioticoli*	WP_023403957	92 (151/164)
28	SS-W, SS-R	628	*Vibrio ezurae*	WP_021713707	69 (101/147)
29	SS-W	349	*Agarivorans* sp. L11	AIY68670	58 (81/139)
30	SH-R	324	*Psychromonas* sp. SP041	WP_025565289	73 (127/173)
31	SS-W	337	*Vibrio ezurae*	WP_021713501	71 (105/148)
32	SS-W	380	*Vibrio breoganii*	WP_017242584	80 (142/178)
33	SH-R	583	*Vibrio cyclitrophicus*	WP_010438368	70 (112/160)
34	SS-W, SS-R	628	*Vibrio ezurae*	WP_021713707	67 (108/161)
35	SH-R	629	*Vibrio variabilis*	GAL29564	65 (115/176)
36	SH-R	614	*Psychromonas* sp. SP041	WP_025564585	74 (126/171)
37	SH-W, SS-W	441	*Agarivorans* sp. L11	AJO61885	97 (132/136)
38	SH-W, SS-W	221	*Vibrio* sp. A9m	BAH79132	96 (155/162)
39	SS-W	324	*Psychromonas* sp. SP041	WP_025565289	75 (128/171)
40	SH-W	358	*Microbulbifer* sp. 6532 A	BAJ62034	63 (71/113)
41	SH-R	629	*Vibrio variabilis*	GAL29564	63 (92/146)
42	SH-W, SH-R	337	*Vibrio ezurae*	WP_021713501	58 (82/141)
43	SS-W, SS-R	302	*Vibrio halioticoli*	WP_039970820	64 (97/151)
44	SH-R	285	*Vibrio cyclitrophicus*	WP_016794789	77 (119/154)
45	SH-W, SH-R	583	*Vibrio cyclitrophicus*	WP_010438368	70 (112/161)
46	SH-R	621	*Psychromonas* sp. SP041	WP_025564997	92 (179/195)
47	SS-W	521	*Vibrio halioticoli*	WP_023403957	93 (150/162)
48	SS-W	323	*Vibrio breoganii*	WP_026026118	77 (107/139)
49	SH-W	358	*Microbulbifer* sp. 6532 A	BAJ62034	61 (99/163)
50	SH-W, SH-R	345	*Vibrio splendidus*	WP_061032701	97 (188/193)

## Discussion

The data obtained in the present study indicate that our controlled feeding experiment could shift the gut bacterial assemblage and ALG gene profiles in two marine gastropod species. A number of 16S rRNA gene phylotypes changed in relative abundance during the rearing experiment; many phylotypes decreased in abundance while an increase was noted for some phylotypes in both SH and SS samples (Fig. 3, Table 1). Six phylotypes increased abundance in the reared SH (phylotypes 26, 66, 72, 82, 156, and 550). These were all close relatives of known alginate-degrading bacterial isolates (Table 1)^20,50–53^. One phylotype, which increased abundance in the reared SS (phylotype 8), was also a relative of known alginate-degrading bacterium (Table 1)^30^. Notably, the reared SH samples had an increased abundance of 16S rRNA phylotypes closely related to the genus *Psychromonas* or *Vibrio* (Fig. 3 and Table 1), which is consistent with the result that the reared SH samples also had ALG gene phylotypes with higher identities to those of *Psychromonas* or *Vibrio* (Fig. 5 and Table 2). We detected enzymatic activities toward alginates in the bacterial fractions isolated from the intestines of the two gastropods with or without rearing (Fig. S3). These results suggested that the rearing experiment enriched some bacterial taxa involved in alginate degradation in their gut microbiota. There were also relatives of alginate-degrading bacteria that decreased in relative abundance after the rearing experiment (Table 1). These bacteria, even if they might actually be alginate degraders, may be less adaptive under the conditions of the present rearing experiment.

The profiles of PL7 ALG gene phylotypes were different between the wild and reared samples. The analyses based on weighted UDs of the phylotypes indicated a higher similarity among individuals within the reared samples compared to those within the wild samples for both host animals (Fig. 4A,B). Together with the finding of the decrease in bacterial members during our rearing experiments (Fig. 1A), these data suggest that on rearing with a single algal species, selected members of bacteria and ALG genes were adaptive to this experimental condition. Many macroalgae are known to reside in the area where the SH and SS individuals used in the present study were captured (data not shown). The wild SH and SS individuals are assumed to live on various types of algae in natural seashore environments^37,54^, leading to a higher gut bacterial richness in wild individuals.

The composition of ALG gene phylotypes was most distinctive between the reared SH and SS samples (Fig. 4A). Actually, the reared SH and SS samples shared no ALG gene phylotype, whereas the wild SH and SS samples shared 6 (Fig. 5; Table 2). The 16S rRNA gene profiles between the reared SH and SS samples were also different (Fig. 2). These data indicate that the functional characteristics of these gastropods as an “incubation pod” are different. Our rearing experiment unexpectedly detected 11 and 4 ALG gene phylotypes otherwise undetected in wild SH and SS, respectively, indicating the significance of our *in vivo* enrichment strategy (Fig. 5, Table 2). The reared-specific ALG genes, as well as “resident” ALG genes such as ALG phylotype 1 in SS samples and phylotype 2 in SH samples, would be involved in the degradation of alginate polymers constituting the brown alga in each gut environment. These ALG genes are promising resources for fermentation procedures using the brown alga. The rearing experiment had impacts on the gut microbiota of marine gastropods and highlighted the ALG genes potentially involved in the degradation of alginate polymers from the target brown alga. The functional validation of the genes will be important in the future.

Change in environmental parameters could affect the type of algae growing in the region, which in turn would impact the gut microbiota of phytophagous or omnivorous aquatic animals.

## Methods

### Gastropods, Alga, and Rearing Experiments

Sea hare (*Dolabella auricularia*) (SH), sea snail (*Batillus cornutus*) (SS), and brown alga (*Ecklonia cava*) samples were all collected in a shallow coastal area (35.1961-35.1976N, 139.5959-139.6012E) of the Nagai area, Yokosuka, Japan. The numbers of the individuals used were: seven for SH wild and reared samples, respectively, and nine and six samples for SS wild and reared samples, respectively. Fresh brown alga was collected at least once a week and kept in one- to two-ton tanks with constant seawater flow during the rearing experiments to feed the gastropods with fresh alga. Rearing experiments were carried out at the National Research Institute of Fisheries Science, Yokosuka, Japan. Animals were individually kept in an aquarium (44 × 33 × 23 cm) containing 26 L seawater. An air stone was placed in each aquarium to provide water circulation and to supply air. Nearshore seawater was pumped up, filtered through sand, and continuously supplied to the aquaria at a rate of 1 L m^−1^. Feeding was started on the same day of the animal collection and continued for two to four weeks. The blade parts of brown alga were washed in tap water prior to feeding. Two to three blades (2‒5 cm wide, 10‒20 cm long) were used to fed SS, whereas the blades were minced using a blender before feeding to SH, which cannot engulf large and hard blades. The rearing water, feces, and remaining food were drained everyday while cleaning the aquaria, and fresh algal food was introduced. Water temperature during the experiment ranged from 14 to 22 °C.

### Sampling of the Gut Contents and Preparation of Bacterial Cell Fraction

Whole body weights were measured on the day of collection and on the last day of the rearing experiment. The shell of SS was broken to take out the soft body. To avoid microbial contamination from the foreign matter attaching to the animal surface, the body surface was washed with tap water followed by rinsing with 70% ethanol. Intestine surrounding midgut gland of SH^55^ and intestine of SS^56^ were dissected on the day of sampling (wild sample) and on the last day of the rearing experiment (reared sample). The gut content from each individual was put into 50 mL tubes with ice-cold 10 mL phosphate-buffered saline (PBS) and three particles of zirconia beads (diameter, 3 mm). The mixture was vortexed for 10 s to separate bacterial cells from the feces matrix and subsequently incubated for 10 min on ice. Two-milliliter of the supernatant was centrifuged at 15,500 × *g* for 3 min. The supernatant was discarded, and the resulting pellet was used as the bacterial cell fraction for metagenomic DNA extraction. For enzymatic activity assay in the fraction, the pellet thus prepared was subjected to two more rounds of washing by using ice-cold PBS described above. The crude extract of the bacterial cell fraction was prepared by sonication.

### Metagenomic DNA Extraction

Metagenomic DNA was extracted from the bacterial cell fraction of the gut samples following the method of Ezaki and Ohkusu^57^ with a minor modification. The bacterial cells described above were mixed with 300 μL lysis solution (91 mM Tris-HCl, 36 mM EDTA, and 0.91% SDS, pH 8.0) and incubated at 70 °C for 10 min. The mixture was subjected to a beads-beating procedure as follows. First, 0.6 g of zirconia beads (diameter, 0.6 mm) was added to the mixture, and the beating step was performed using a multi-beads shocker (Yasui-kikai, Osaka, Japan) at 2,500 rpm for 3 min in 2 mL tubes. Then, 400 μL of lysis solution was added to the homogenates followed by incubation at 70 °C for 10 min. The metagenomic DNA in the supernatant was purified with phenol-chloroform-isoamyl alcohol (25:24:1) extraction followed by ethanol precipitation.

Each DNA sample was further purified using AM Pure XP (Beckman coulter) and NucleoSpin gDNA Clean-up XS (Macherey-Nagel). The final DNA concentration was measured using a Quant-iT PicoGreen dsDNA Assay kit (Life Technologies).

### PCR Amplification, Pyrosequencing, and Annotation of 16S rRNA Gene

The V1-V2 region of the 16S rRNA gene was amplified with a universal primer set with some modifications, accompanied by 454 pyrosequencing adaptors (underlined) and the common MID tag sequences (as indicated in bold). The primer sequences were as follows: M27F (5′-CCATCTCATCCCTGCGTGTCTCCGACTCAG-[common MID]- AKWGTTTGATCMTGGCTCAG-3′) as the forward primer and M338R (5′-CCTATCCCCTGTGTGCCTTGGCAGTCTCAG CTGCWGCCWYCCGTAGRWGT-3′) as the reverse primer. PCR amplification of 16S rRNA gene was performed in a 25 μL mixture containing 1 ng of metagenomic DNA, 12.5 μL of PrimeSTAR Max DNA polymerase (Takara), and 0.4 µM forward and reverse primers with the following PCR conditions: 5 min of initial denaturation at 98 °C, 25 cycles of 10 s of denaturation at 98 °C, 15 s of annealing at 49 °C, and 5 s of extension at 72 °C, followed by a 5 min final extension at 72 °C. The purification, quantification, pyrosequencing, filtering, and annotation of the 16S rRNA gene amplicons were carried out as described previously^58^.

### PCR Amplification of PL7 Alginate Lyase Genes

Two conserved regions of PL7 ALG, the A3 and A4 strands, were employed as target sites for the construction of degenerate primers. For common nucleotide sequences within, we referred to 16 sequences of characterized PL7 enzymes in the CAZy database^59^ and 70 Pfam 08787 sequences^60^. Because the sequences of the forward primer are relatively less conserved, two sets of forward primers were designed, and the mixture of those sets was used upon amplification. The forward primers were 5′-TAYISIMGITCIGARYTIMGNG-3′ (ALG-f1) and 5′-TAYISIMGIAGIGARYTIMGNG-3′ (ALG-f2). The reverse primer was 5′-RTANNNICCIGCYTTRAARTA-3′ (ALG-r).

ALG gene partial fragments were PCR amplified from gut metagenomic DNA in a 25 μL mixture (total volume) containing 1 ng template DNA, 0.67 µM each primer, and 12.5 µL Premix Taq Hot Start Version (TaKaRa-Bio Inc., Otsu, Japan) with the following PCR conditions: 1 min of initial denaturation at 94 °C, 35 cycles of 20 s denaturation at 94 °C, 20 s annealing at 35 °C, and 48 s extension at 72 °C, followed by a 7 min final extension at 72 °C.

### Construction of the Alginate Lyase Gene Clone Library

Because PCR products potentially containing ALG gene amplicons were estimated to be within the range of 300‒650 bp, the products in this range were purified using a Gel Extraction Kit (Qiagen, Hilden, Germany). The purified fragments were subjected to A-tailing and ligated to a pGEM-T Easy Vector (Promega) by TA cloning. The ligation products were directly used for transformation of ECOS competent *Escherichia coli* JM109 (Nippon gene). The positive clones were primarily selected with blue-white selection on Luria-Bertani (LB) agar plates containing 50 mg L^−1^ ampicillin, 0.5 mM isopropyl-β-D-thiogalactopyranoside (IPTG), and 40 mg L^−1^ 5-bromo-4-chloro-3-indoyl-β-D-galactopyranoside (X-gal). The insert DNA fragments in the pGEM-T Easy Vector were amplified by colony PCR using M13 primers. Forty-eight clones were randomly selected per each library from the clones that passed these steps. The insert fragments of the clones were sequenced by the Sanger method and analyzed by BLASTP searches, as described in the next section. When the number of clones with any similarity to known ALGs in the database was less than 20 among the 48 clones, 20‒61 clones were additionally sequenced.

### Filtering of Alginate Lyase Genes

The deduced amino acid sequences of the cloned fragments in all six frames were subjected to two filtering steps as follows: (i) frames with any stop codon were excluded, and subsequently, (ii) BLASTP searches were carried out against the nr database to exclude sequences not likely to have originated from an ALG gene. The amino acid sequences that did not contain known or putative ALGs in NCBI database were discarded.

### Phylotype Clustering

The sequences that passed the above-mentioned filtering steps were aligned with MAFFT^61^ and manually trimmed to equal lengths. The sequences of the 16S rRNA gene and ALG genes were adjusted to 280 bp and 95 aa, respectively. Any sequences shorter than the lengths were excluded. Subsequently, we employed UPARSE^62^ to cluster the 16S rRNA and ALG gene sequences into phylotypes defined by 97% nucleotide and 90% amino acid identity, respectively. Representative sequences of each phylotype were chosen from the reads found at least two times. The Chao1^63^ and Shannon indices^64^ were estimated for each of the defined phylotypes to evaluate 16S rRNA gene and ALG richness and diversity in the gut microbiota.

### UniFrac Distance Analysis

Fast UniFrac^47^ was used to calculate UniFrac distances and their principle coordinates among the phylotypes. For the unweighted UniFrac distance analysis of 16S rRNA genes, to avoid bias due to differences in sequencing depth, 4,000 reads were randomly sampled and used for the analysis. Phylogenetic trees for the representative sequences of each phylotype were constructed using the neighbor-joining method.

### Nucleotide Sequence

The 454 sequence runs were deposited in DDBJ (DNA Data Bank of Japan) Sequence Read Archive (DRA) under accession no. DRA005428. The representative sequences of alginate lyase gene phylotypes found in the samples of wild SH, reared SH, wild SS and reared SS were deposited in DDBJ under accession numbers LC186928 to LC186944, LC188888 to LC188910, LC188911 to LC188934, and LC188935 to LC188949, respectively.

### Statistical Analysis

All statistical tests were performed with SigmaPlot 12 software (Systat Software). Because normality tests failed for some values of richness and diversity indices and UniFrac distances and the values of relative abundance of each phylotype do not follow normal distribution in principle, comparison of those values were carried out using non-parametric Mann-Whitney U tests at the α = 0.05 significance level.

## Supplementary information

Dataset 1
