# Supplementary material for: Enrichment of bacteria and alginate lyase genes potentially involved in brown alga degradation in the gut of marine gastropods
Source: Sci Rep. 2019 Feb 14;9:2129. doi: 10.1038/s41598-018-38356-y (PMC6375959; doi:10.1038/s41598-018-38356-y)
Supplement: Supplementary file 1 — Dataset 1 [file 41598_2018_38356_MOESM1_ESM.pdf]

**Supplementary Material**

# Enrichment of bacteria and alginate lyase genes potentially involved in brown alga degradation in the gut of marine gastropods

M. Ito, K. Watanabe, T. Maruyama, T. Mori, K. Niwa, S. Chow, H. Takeyama

**Table S1** Summary of descriptive data in this study. [a] SH, sea hare ; SS, sea snail. [b] NA, not analyzed.

| Sample type[a] | | Sample no. | Captured date | Dissection date | Initial weight | Final weight | Weight change | Amplicon pyrosequencing analysis of 16S rRNA genes | | | | | | | Clone library analysis of PL7 ALG genes | |
|---|---|---|---|---|---|---|---|---|---|---|---|---|---|---|---|---|
| | | | | | (gram, wet weight) | | | No. of reads | with all reads | | | with 4,000 reads | | | | |
| | | | | | | | | | No. of phylotypes | Richness (Chao1) | Diversity (Shannon) | No. of phylotypes | Richness (Chao1) | Diversity (Shannon) | No. of cloned sequences | No. of positive sequences |
| SH | Wild | 1 | 111129 | | NA[b] | | - | 24907 | 821 | 823.4 | 3.96 | 235 | 248.3 | 3.44 | 85 | 37 |
| | | 2 | | | NA[b] | | | 8263 | 193 | 271.8 | 2.92 | 130 | 169 | 2.79 | 48 | 38 |
| | | 3 | 130416 | | 234 | | - | 12470 | 208 | 281 | 2.76 | 120 | 203.2 | 2.65 | 48 | 42 |
| | | 4 | 130515 | | 454 | | - | 11026 | 140 | 175.4 | 2.55 | 73 | 82.2 | 2.46 | 48 | 31 |
| | | 5 | | | 289 | | | 8340 | 216 | 313.9 | 2.75 | 119 | 160.4 | 2.58 | 48 | 36 |
| | | 6 | | | 229 | | | 7805 | 136 | 179 | 2.64 | 101 | 158.3 | 2.61 | 48 | 31 |
| | | 7 | | | 329 | | | 8350 | 83 | 140 | 2.48 | 67 | 101.2 | 2.39 | 48 | 33 |
| | Reared | 1 | 111129 | 111229 | 256 | 220 | -36 | 7809 | 143 | 169.4 | 2.18 | 91 | 99.6 | 2.09 | 48 | 34 |
| | | 2 | | | 374 | 338 | -36 | 4035 | 81 | 112.6 | 1.91 | 76 | 101.7 | 1.84 | 48 | 46 |
| | | 3 | | | 404 | 313 | -91 | 11239 | 111 | 130.5 | 2.22 | 78 | 101.1 | 2.16 | 48 | 38 |
| | | 4 | 130501 | 130515 | 551 | 477 | -74 | 9104 | 74 | 91.1 | 2.72 | 54 | 58.5 | 2.63 | 48 | 33 |
| | | 5 | | | 381 | 329 | -52 | 7502 | 70 | 74.5 | 2.63 | 60 | 86 | 2.58 | 48 | 38 |
| | | 6 | 130416 | 130515 | 335 | 343 | 8 | 5151 | 62 | 73.1 | 2.67 | 54 | 63.4 | 2.6 | 48 | 42 |
| | | 7 | | | 225 | 231 | 6 | 6916 | 44 | 59.6 | 1.96 | 42 | 66 | 1.96 | 48 | 31 |
| SS | Wild | 1 | 111207 | | 73 | | - | 9067 | 146 | 234.7 | 2.12 | 90 | 164.4 | 1.94 | 104 | 14 |
| | | 2 | | | 80 | | | 8737 | 41 | 44.5 | 1.30 | 28 | 29.3 | 1.14 | 83 | 31 |
| | | 3 | 130416 | | 152 | | - | 15112 | 137 | 171.1 | 1.57 | 67 | 113.2 | 1.42 | 108 | 0 |
| | | 4 | | | 164 | | | 11153 | 110 | 182.1 | 1.89 | 60 | 100.6 | 1.72 | 48 | 30 |
| | | 5 | | | 151 | | | 11771 | 124 | 217.2 | 1.75 | 61 | 72.9 | 1.55 | 96 | 23 |
| | | 6 | 130515 | | 95 | | - | 8973 | 116 | 158 | 2.47 | 84 | 126 | 2.31 | 48 | 34 |
| | | 7 | | | 129 | | | 8686 | 107 | 144 | 2.52 | 72 | 101.5 | 2.38 | 48 | 42 |
| | | 8 | | | 110 | | | 9069 | 180 | 243 | 2.31 | 98 | 125.6 | 2.13 | 96 | 16 |
| | | 9 | | | 133 | | | 8716 | 116 | 214 | 2.30 | 67 | 77.9 | 2.13 | 48 | 39 |
| | Reared | 1 | 111129 | 111229 | 164 | 169 | 5 | 9847 | 40 | 47 | 1.75 | 31 | 41.5 | 1.66 | 109 | 5 |
| | | 2 | | | 87 | 92 | 5 | 7729 | 30 | 37.5 | 1.77 | 23 | 25 | 1.58 | 48 | 43 |
| | | 3 | 130416 | 130515 | 143 | 157 | 14 | 8565 | 39 | 43.7 | 1.59 | 29 | 36 | 1.35 | 48 | 39 |
| | | 4 | | | 151 | 173 | 22 | 7518 | 40 | 55.2 | 1.56 | 30 | 46.5 | 1.42 | 83 | 43 |
| | | 5 | | | 145 | 143 | -2 | 8142 | 41 | 42 | 1.78 | 33 | 38.1 | 1.57 | 48 | 29 |
| | | 6 | | | 208 | 209 | 1 | 8361 | 43 | 48.5 | 1.41 | 25 | 26 | 1.18 | 48 | 35 |

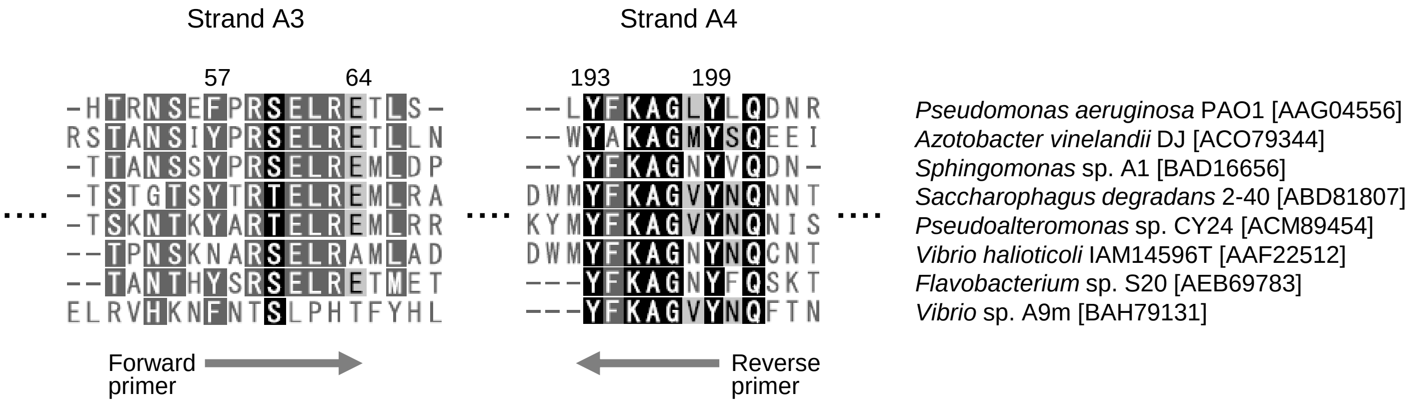


**Fig. S1** Amino acid sequence region used to design degenerate primers for amplification of PL7 family alginate lyase gene partial sequences. The arrows indicate the regions and directions of the primers. The numbers on the alignment show the corresponding amino-acid position of the PL7 alginate lyase of *Pseudomonas aeruginosa* strain PAO1 (accession no. AAG04556).

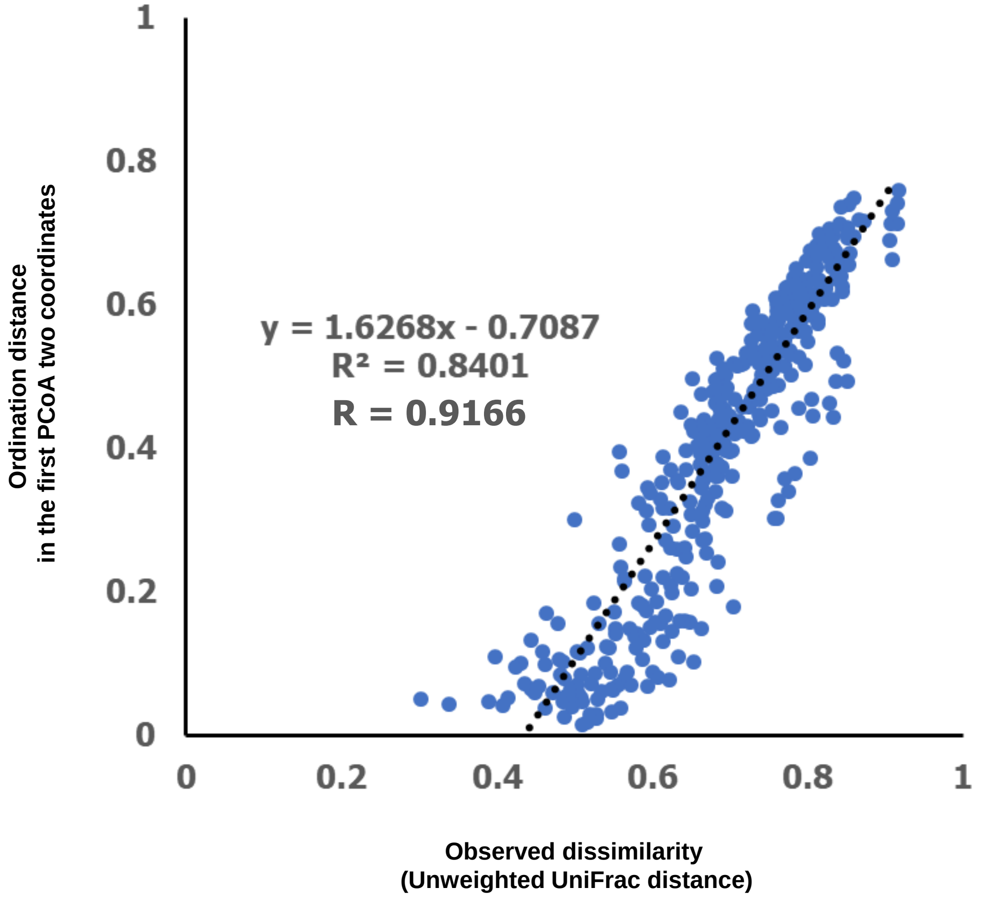


**Fig. S2** Shepard diagram of the data from Fig. 2A. The scatter of points clearly shows the correlation between the unweighted UniFrac distances and the inter-sample distances in the first two PCoA coordinates ($R > 0.9$), suggesting that the unweighted UniFrac distances are well preserved by the first two coordinates of the PCoA (43.5% in total).

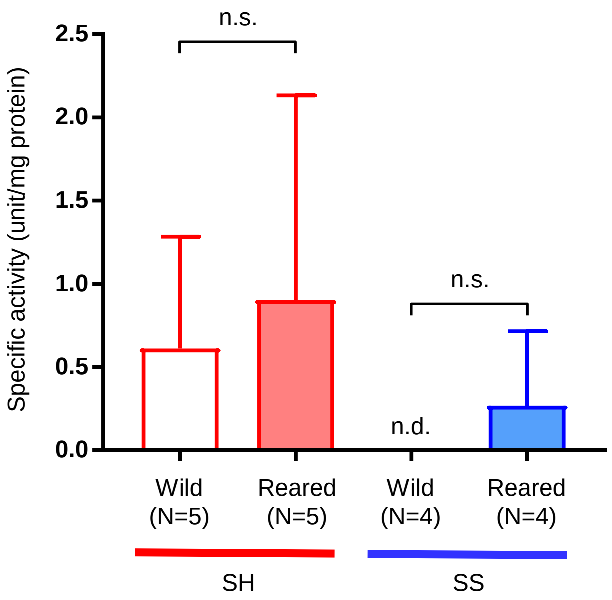


**Fig. S3** Alginate degradation activity in the gut microbiota of the sea hare (SH) *Dolabella auricularia* and the sea snail (SS) *Batillus cornutus.* The crude extract of the bacterial cell fraction in their gut was prepared as described in "Sampling of the Gut Contents and Preparation of Bacterial Cell Fraction" in Methods section and was used for the activity assay as described in the ref 29.One unit of specific activity was defined as the activity that produces reducing sugar equivalent to 1 μmol of uronic acid monomer (galacturonic acid) per min. Error bars indicate standard deviation. The values were statistically compared using t-test (α=0.05). n.s., not significant; n.d., not detected; N, the number of the biological replicate.
